# Proteomic analysis reveals metabolic and regulatory systems involved in the syntrophic and axenic lifestyle of *Syntrophomonas wolfei*

**DOI:** 10.3389/fmicb.2015.00115

**Published:** 2015-02-11

**Authors:** Jessica R. Sieber, Bryan R. Crable, Cody S. Sheik, Gregory B. Hurst, Lars Rohlin, Robert P. Gunsalus, Michael J. McInerney

**Affiliations:** ^1^Department of Botany and Microbiology, University of OklahomaNorman, OK, USA; ^2^Department of Geological Sciences, University of MichiganAnn Arbor, MI, USA; ^3^Chemical Sciences Division, Oak Ridge National LaboratoryOak Ridge, TN, USA; ^4^Department of Microbiology, Immunology, and Molecular Genetics, University of California, Los AngelesLos Angeles, CA, USA

**Keywords:** syntrophy, *Syntrophomonas wolfei*, interspecies electron transfer, reverse electron transfer, hydrogen, methanogenesis

## Abstract

Microbial syntrophy is a vital metabolic interaction necessary for the complete oxidation of organic biomass to methane in all-anaerobic ecosystems. However, this process is thermodynamically constrained and represents an ecosystem-level metabolic bottleneck. To gain insight into the physiology of this process, a shotgun proteomics approach was used to quantify the protein landscape of the model syntrophic metabolizer, *Syntrophomonas wolfei*, grown axenically and syntrophically with *Methanospirillum hungatei*. Remarkably, the abundance of most proteins as represented by normalized spectral abundance factor (NSAF) value changed very little between the pure and coculture growth conditions. Among the most abundant proteins detected were GroEL and GroES chaperonins, a small heat shock protein, and proteins involved in electron transfer, beta-oxidation, and ATP synthesis. Several putative energy conservation enzyme systems that utilize NADH and ferredoxin were present. The abundance of an EtfAB2 and the membrane-bound iron-sulfur oxidoreductase (Swol_0698 gene product) delineated a potential conduit for electron transfer between acyl-CoA dehydrogenases and membrane redox carriers. Proteins detected only when *S. wolfei* was grown with *M. hungatei* included a zinc-dependent dehydrogenase with a GroES domain, whose gene is present in genomes in many organisms capable of syntrophy, and transcriptional regulators responsive to environmental stimuli or the physiological status of the cell. The proteomic analysis revealed an emphasis on macromolecular stability and energy metabolism by *S. wolfei* and presence of regulatory mechanisms responsive to external stimuli and cellular physiological status.

## Introduction

The metabolic cooperation called syntrophy is a thermodynamically-based interaction between two or more microorganisms, which must rely on each other to maintain pool sizes of exchanged metabolites at sufficiently low concentrations so that the overall catabolic conversion is thermodynamically favorable (Schink and Stams, 2006; McInerney et al., 2008). Syntrophy is an essential intermediary step in the anaerobic degradation of natural polymers such as polysaccharides, proteins and lipids where syntrophic associations are needed to convert the products of fermentative microorganisms, fatty acids, alcohols and aromatic compounds, to methanogenic growth substrates acetate, H_2_ and formate (McInerney et al., 2008; Stams and Plugge, 2009). Thus, syntrophic fatty acid metabolism accounts for much of the carbon flux and methane production in methanogenic environments (McInerney et al., 2008). Despite the ubiquity of syntrophy processes in anoxic environments, little is known about mechanisms by which syntrophic consortia regulate their metabolism.

*S. wolfei* is a metabolic specialist that syntrophically metabolizes a very limited number of fatty acids from four to eight carbons in length to acetate, H_2_ and formate (McInerney et al., 1979, 1981; Beaty and McInerney, 1987). It can grow axenically on unsaturated fatty acids such as crotonate by oxidizing part of the molecule to acetate and reducing to remainder to the respective saturated fatty acid (Beaty and McInerney, 1987; Amos and McInerney, 1990). However, to oxidize saturated fatty acids, *S. wolfei* requires the presence of a suitable H_2_- and/or formate-consuming partner (i.e., a methanogen) to maintain H_2_ and formate at sufficiently low levels so that saturated fatty acid degradation is thermodynamically favorable (Schink, 1997). This allows *S. wolfei* to reoxidize its reduced electron carriers by forming H_2_ and formate rather than by using the unsaturated fatty acid as an electron acceptor. Thus, the interaction between *S. wolfei* and *M. hungatei* during growth on crotonate is beneficial to each species but not obligatory as when *S. wolfei* grows syntrophically with *M. hungatei* on butyrate.

A critical physiological feature of *S. wolfei* during syntrophic growth on saturated fatty acids is the requirement for reverse electron transfer to produce H_2_ (E′ of −261 mV at 1 Pa H_2_) and formate (E′ of −258 mV at 1 μM formate) from electrons generated in the oxidation of acyl-CoA intermediates to their respective enoyl-CoA intermediates (E′ of −10 mV) (Sato et al., 1999). This redox reaction is energetically unfavorable (ΔE′ of ~ −250 mV) and requires energy input to drive the reaction forward. The use of inhibitors showed that a chemiosmotic gradient is required for hydrogen production from butyrate (Wallrabenstein and Schink, 1994). A reverse quinone loop involving a membrane-bound, electron transfer flavoprotein (EtfAB):menaquinone oxidoreductase and either a membrane-bound hydrogenase or formate dehydrogenase has been hypothesized to use the proton motive force to produce H_2_ or formate, respectively, from electrons derived from the oxidation of butyryl-CoA (Schink, 1997; Sieber et al., 2012; Schmidt et al., 2013). The reverse quinone loop model for syntrophic reverse electron transfer is supported by the more than 100-fold higher expression of a membrane-bound hydrogenase, *hyd2* (Sieber et al., 2014), and the presence of a membrane-bound formate dehydrogenase, Fdh2 (Schmidt et al., 2013) when *S. wolfei* is grown with *M. hungatei* on butyrate. In addition, a membrane-bound, iron-sulfur protein that may function as an EtfAB:menaquinone oxidoreductase and EtfAB2 were detected in the *S. wolfei* proteome (Schmidt et al., 2013). However, the genome of *S. wolfei* contains other possibilities for reverse electron transfer including the Fix system and a bifurcating, butyryl-CoA dehydrogenase (Bcd):EtfAB1 (Sieber et al., 2010).

Unlike organisms capable of syntrophy such as sulfate and iron reducers, *S. wolfei* cannot use alternative electron acceptors for growth (Sieber et al., 2010). The limited metabolic potential of *S. wolfei* makes it an ideal model organism for identifying the essential machinery of syntrophy, but makes it difficult to use genetic approaches to identify syntrophic processes. The genomes of *S. wolfei* (Sieber et al., 2010) and *M. hungatei* (NCBI Reference Sequence: NC_007796) have been recently sequenced and annotated, which has opened the investigation of syntrophy to high-throughput analyses. Genomic analyses of *S. wolfei* revealed metabolic specialization and nutritional self-sufficiency consistent with its limited metabolic potential (Sieber et al., 2010). Thus, *S. wolfei* appears to be genetically “hard-wired” for syntrophy. As a metabolic specialist that survives on reactions close to thermodynamic equilibrium, we hypothesize that *S. wolfei* is physiologically adapted to fatty acid metabolism and hydrogen and/or formate production and the shift from axenic to syntrophic growth involves a limited number of enzyme systems rather than the large global changes in gene expression that have been detected with sulfate reducers (Meyer et al., 2013a,b). In this study, we used whole cell proteomic analyses of *S. wolfei* grown alone and in coculture with *M. hungatei* grown axenically to identify the major metabolic systems used for axenic and syntrophic lifestyles.

## Materials and methods

### Organisms and growth conditions

Pure cultures of *Syntrophomonas wolfei* (DSM 2245B) (McInerney et al., 1981) and cocultures of *S. wolfei* with *Methanospirillum hungatei* strain JF1 (ATCC 27890) were grown anaerobically as described previously (McInerney et al., 1979). *S. wolfei* pure cultures were grown in medium with 20 mM crotonate. *S. wolfei*-*M. hungatei* cocultures were grown in medium with 20 mM crotonate or 20 mM butyrate. Media were prepared using a modified Balch technique (Balch and Wolfe, 1976). All cultures were grown in 75 ml volumes in 160 ml serum bottles in triplicate. The headspace was N_2_/CO_2_ (80:20 v/v). All cultures were incubated at 37°C without shaking. Culture purity was checked daily by microscopic examination and inoculation of a thioglycolate medium. Cultures were transferred repeatedly until the growth rate and/or methane production rate among the replicates of a given growth condition were nearly equal. Cells were harvested at 50% substrate loss, which was in the late exponential phase of growth as determined by change in absorbance for pure cultures and methane production for cocultures.

Growth was monitored by measuring optical density at 600 nm. One milliliter of samples were taken daily to measure substrate depletion and product formation. Methane formation by cocultures was measured by daily headspace analysis. Methane was measured by gas chromatography with a flame ionization detection equipped with Poropak Q, 80/100 column (6 feet × 1/8 inch) (Supelco, Bellefonte, PA). The injector temperature was set at 100°C, the column at 100°C and the detector at 125°C. Helium was used as a carrier gas.

The concentrations of crotonate, butyrate, and acetate were determined by high performance liquid chromatography with a Prevail Organic acid column (250 by 4.6 mm; particle size 5 μm; Alltech Inc, Deerfield, Ill.) at a flow rate of 1 ml/min. The isocratic mobile phase consisted of 25 mM KH_2_PO_4_ (pH 2.5) to measure acetate concentrations. A mobile phase of 60% (v/v) KH_2_PO_4_ (25 mM, pH 2.5):40% (v/v) acetonitrile was used to quantify crotonate and butyrate. The UV absorbance detector was set at 210 nm to detect acetate and butyrate, and 254 nm for crotonate.

### Sample preparation

Duplicate cultures of the three growth conditions, e.g., *S. wolfei* pure culture on crotonate, *S. wolfei*-*M. hungatei* on crotonate and *S. wolfei*-*M. hungatei* on butyrate, were harvested at 50% substrate loss by centrifugation (14,300 × G, 20 min, 4°C) and processed separately shotgun proteomics analysis. Cell pellet wet weights were 90 mg and 73 mg for *S. wolfei* pure cultures, 105 and 79 mg for *S. wolfei*-*M. hungatei* cocultures on crotonate, and 61 and 67 mg for *S. wolfei*-*M. hungatei* coculture on butyrate. Cell pellets were processed by generally following a protocol optimized for measurements of small bacterial samples (Thompson et al., 2008). Cell pellets were lysed and proteins denatured by incubating each cell pellet overnight at 37°C in 250–400 μL of 6 M guanidine and 10 mM dithiothreitol (DTT) (larger volumes used for larger cell pellets). Lysates were cooled to ambient temperature, and diluted with 50 mM Tris with 10 mM CaCl_2_ to decrease the guanidine concentration to ~1 M. Ten micrograms of trypsin (sequencing grade, Promega, Madison WI) was added to each lysate, followed by a 5-h incubation at 37°C. An additional 10 μg trypsin was added, followed by a further overnight incubation at 37°C. Remaining disulfide bonds were reduced by adding additional DTT to a final concentration of 10 mM and incubation for 1 h at 37°C. Desalting was performed using reverse-phase solid-phase extraction cartridges (Sep-Pak Lite C18, Waters, Milford MA), with final elution using 0.1% formic acid in acetonitrile. Solvent transfer to aqueous 0.1% formic acid was performed by vacuum centrifugation, with final volume adjusted to 150 μl. Particulates and remaining cellular debris were removed by centrifugation through 0.45 μm pore filters (Ultrafree-MC, Millipore, Billerica MA). Samples were frozen at −80°C until further use.

### LC-MS-MS analysis

Tryptic peptide mixtures were analyzed by two-dimensional liquid chromatography/tandem mass spectrometry (2D LC-MS-MS), using the MudPIT approach (Washburn et al., 2001; Wolters et al., 2001) implemented as previously described in further detail (Hervey et al., 2009). Two LC-MS-MS analyses were performed on the tryptic digest from each cell pellet. Thus, for each growth condition, two technical replicates were analyzed for each of the two biological replicates. Aliquots (50 μl) were loaded via a pressure cell (New Objective, Woburn MA) onto a “back” column fabricated from 150 μm internal diameter (ID) fused silica tubing (Polymicro Technologies, Phoenix AZ) packed with a ~4 cm long bed of reverse-phase chromatographic phase (Jupiter C18, 3 μm particle size, Phenomenex, Torrance CA) upstream of a ~4 cm bed of strong cation exchange material (5 μm particle size SCX, Phenomenex).

After sample loading, the back column was attached via a filter union (Upchurch Scientific, Oak Harbor WA) to a “front” analytical column fabricated from a 100 μm ID PicoTip Emitter (New Objective), packed with a ~14 cm bed of reverse-phase material (Jupiter C18, 3 μm particle size, Phenomenex). Two-dimensional LC was performed via 12 step gradients of increasing salt (ammonium acetate) concentration, with the eluted peptides from each strong cation exchange step subsequently resolved via a separate reverse-phase gradient (Accela HPLC, ThermoScientific, San Jose CA). The LC eluent was interfaced via a nanospray source (Proxeon, Odense, Denmark) with a linear-geometry quadrupole ion trap mass spectrometer (LTQ-XL, ThermoScientific, San Jose CA). Data acquisition was performed in data-dependent mode under the control of XCalibur software. Up to five tandem mass spectra were acquired from the most abundant parent ions in full-scan mass spectra; dynamic exclusion was enabled with a repeat count of 1 and duration of 60 s.

### Proteomics data analysis

Peptide identifications were obtained from tandem mass spectra using Sequest software (version 27) (Eng et al., 1994), and protein identifications were compiled from peptide identifications using DTASelect (version 1.9) (Tabb et al., 2002). A multiple-species protein FASTA file was constructed from individual FASTA files for *S. wolfei* subspecies *wolfei* Göttingen, *M. hungatei* JF-1, and *Syntrophus aciditrophicus* strain SB downloaded from the DOE Joint Genome Institute website. The sequence-reversed analog of each protein sequence was appended to the FASTA file to allow estimation of the false discovery rate of peptide identification (Moore et al., 2002; Elias and Gygi, 2007). Sequences of 36 common contaminant proteins were also appended to the FASTA file. The complete FASTA file contained 18006 entries. Peptide identifications were retained for XCorr ≥1.8 (charge state *z* = 1), ≥2.5 (*z* = 2), or ≥3.5 (*z* = 3), with DeltaCN ≥0.08. Protein identifications required identification of two peptides, or a single peptide in two different charge states. The false discovery rate for peptides was generally ≤1%. Estimates of protein abundance were calculated using normalized spectral abundance factors (NSAF) (Zybailov et al., 2006).

Non-metric multidimensional scaling (NMDS) was performed using Bray-Curtis dissimilarities of NSAFs from different growth conditions. Bray-Curtis utilizes a shared presence absence matrix to score similarity, which is why it has been well used in ecology to assess the similarity of two communities. Rare or missing proteins were included within the analysis as these may have biological significance, with the caveat that if a protein was detected in only in a single replicate of a treatment it was left out. NMDS was calculated using the vegan package (Oksanen et al., 2011) as implemented in R (R Development Core Team, 2011).

## Results and discussion

### Proteomic overview of *S. wolfei*

The repertoire of proteins involved in syntrophic and axenic growth of *S. wolfei* was characterized by growing *S. wolfei* in pure culture on crotonate and in coculture with *M. hungatei* on either crotonate or butyrate (Supplemental Figure 1). The genome of *S. wolfei* contains 2574 protein encoding genes (Sieber et al., 2010) and the proteomic analysis detected a total of 790 proteins among the three growth conditions. Of these, 106 are proteins without a known function (Supplemental Data Set 1).

**Figure 1:**
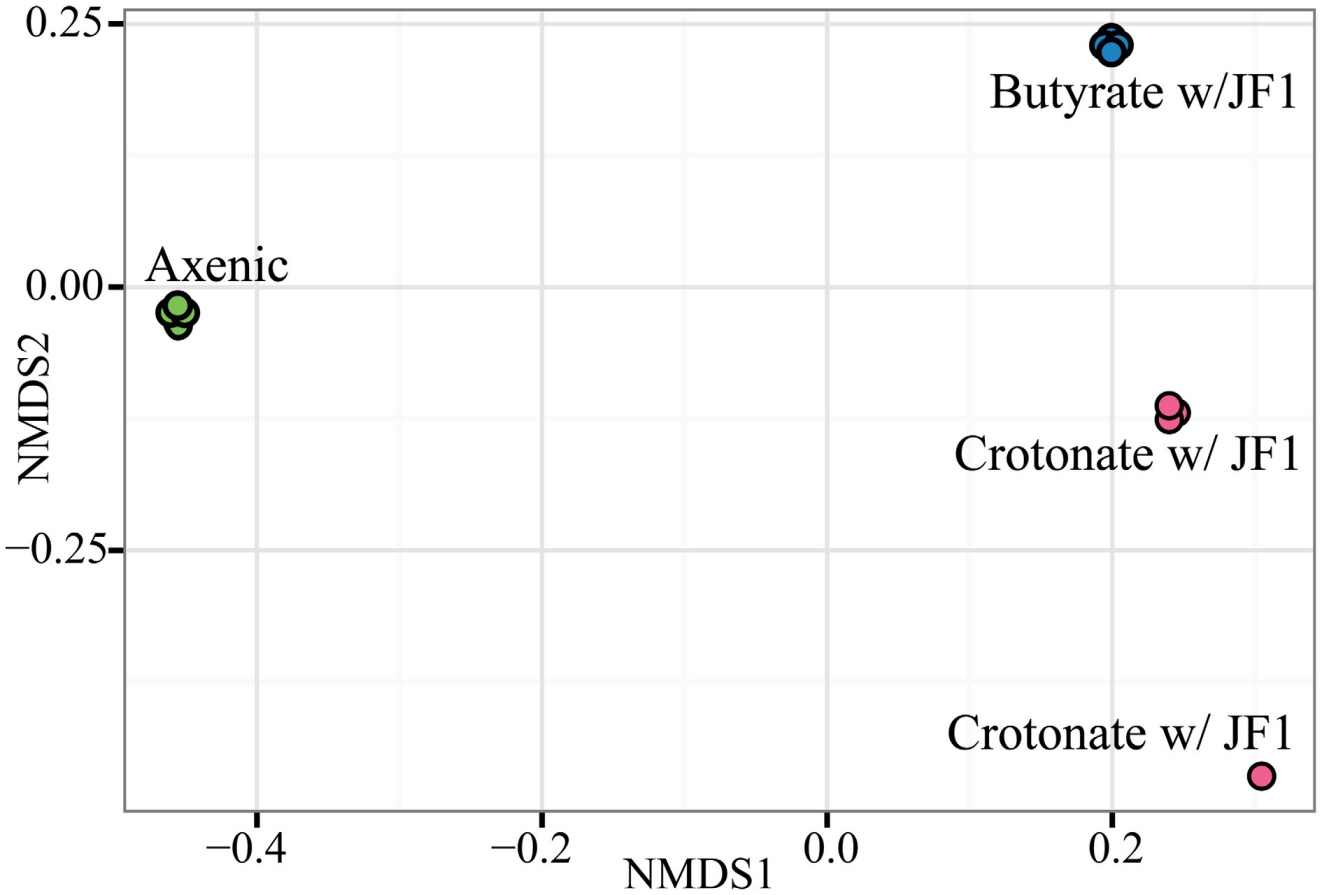
**Non-metric multidimensional scaling of the *S. wolfei* protein profiles obtained from each technical replicate for each duplicate culture grown under the three conditions**. Symbols: green circle, *S. wolfei* pure culture on crotonate; pink circles, *S. wolfei-M. hungatei* coculture on crotonate; blue circles, *S. wolfei-M. hungatei* coculture on butyrate.

NMDS ordination using distance metrics that include presence/absence and abundance revealed protein abundance patterns between axenic and syntrophic growth conditions were highly reproducible among the replicates of a given growth condition (Figure 1). Only one technical replicate of a *S. wolfei-M. hungatei* coculture grown on crotonate differed from the other three and this was driven primarily by low protein recovery. Nonetheless, the ordination value of this replicate was much closer to those for the other crotonate-grown coculture replicates than to those of the other growth conditions. These results allowed us to evaluate the contributions of multiple hydrogenases, electron transfer flavoproteins (Etf), formate dehydrogenases and paralogous enzymes involved in fatty acid metabolism to axenic and syntrophic lifestyles. NMDS ordination analysis showed that the protein pattern of *S. wolfei* as represented by the NSAF was different when *M. hungatei* was present.

**Figure 2:**
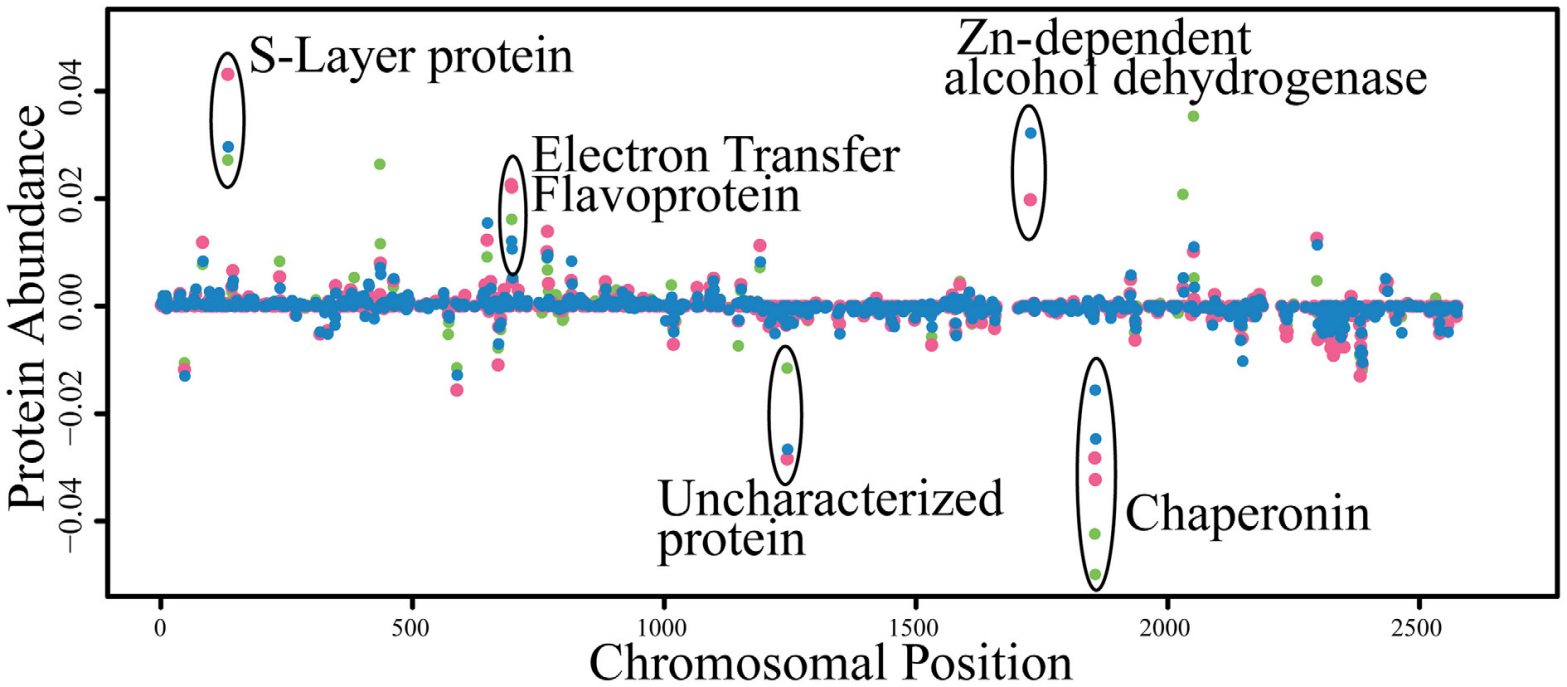
**Abundance of peptides detected from each condition mapped by genomic location**. Proteins encoded by genes on the lagging strand are represented as negative abundance. Symbols: green circle, *S. wolfei* pure culture on crotonate; pink circles, *S. wolfei-M. hungatei* coculture on crotonate; blue circles, *S. wolfei-M. hungatei* coculture on butyrate.

The number of proteins detected between the three growth conditions is compared in Supplemental Figure 2. Three hundred and ninety-one proteins were detected under all three conditions of which 113 appear to be constitutively present (i.e., less than 0.5-fold change among all conditions; Supplementary Table 1). Four open reading frames previously predicted to be pseudogenes (Swol_0818, Swol_1580, Swol_2335, and Swol_2574) were found to be protein encoding. Remarkably, the protein abundance, as represented by NSAF of most proteins changed very little between the pure and coculture growth conditions (Figure 2).

### Highly abundant proteins during all growth conditions

Highly abundant proteins were those involved in major pathways and processes within the cell. Nine proteins had an NSAF greater than 0.01 under all three growth conditions including two chaperonins (GroEL and GroES), one small heat shock protein (Swol_0588 gene product), two paralogs of the DNA-binding proteins HU, rubrerythrin (a protein employed during oxidative stress), two transcription factors, and the Swol_0133 gene product, annotated as a putative copper amine oxidase (Table 1). The Swol_0133 gene product is a predicted cytoplasmic protein although a role in cell envelope function was recently proposed (Schmidt et al., 2013). Other abundant proteins had annotated functions involved in beta-oxidation, electron transfer and energy production (Table 1). GroEL and GroES were among the most abundant proteins in *Escherichia coli* (See datasets in Lu et al., 2007; Mancuso et al., 2012). The types of abundant proteins detected emphasize the importance of macromolecular stability and energy metabolism to *S. wolfei*.

**Table 1:** **The most abundant peptides detected in each condition**.

**Locus tag**	**Gene description**	**NSAF of peptides detected**		
		**Crotonate**	**Crotonate with *M. hungatei***	**Butyrate with *M. hungatei***
Swol_0047	Transcriptional regulator, AbrB family	0.010 ± 0.002	0.011 ± 0.004	0.011 ± 0.001
Swol_0083	DNA-binding protein HU	0.008 ± 0.0004	0.011 ± 0.003	0.007 ± 0.002
Swol_0133	Copper amine oxidase	0.027 ± 0.002	0.042 ± 0.010	0.026 ± 0.003
Swol_0435	3-hydroxybutyryl-CoA dehydrogenase	0.026 ± 0.002	0.002 ± 0.000	0.006 ± 0.001
Swol_0436	Coenzyme A transferase	0.011 ± 0.0004	0.008 ± 0.002	0.005 ± 0.001
Swol_0588	Small heat shock protein	0.011 ± 0.001	0.015 ± 0.004	0.011 ± 0.002
Swol_0648	DNA-binding protein HU	0.009 ± 0.0003	0.012 ± 0.002	0.008 ± 0.001
Swol_0670	Rubrerythrin	0.008 ± 0.001	0.010 ± 0.003	0.006 ± 0.001
Swol_0696	Electron transfer flavoprotein β-subunit	0.022 ± 0.003	0.022 ± 0.005	0.010 ± 0.001
Swol_0697	Electron transfer flavoprotein α-subunit	0.016 ± 0.001	0.021 ± 0.004	0.009 ± 0.001
Swol_0767	Phosphate acetyltransferase	0.009 ± 0.0003	0.010 ± 0.001	0.008 ± 0.0003
Swol_0768	Acetate kinase	0.007 ± 0.001	0.013 ± 0.002	0.008 ± 0.001
Swol_1190	Molybdenum-pterin-binding protein	0.007 ± 0.001	0.011 ± 0.003	0.007 ± 0.001
Swol_1244	Polyhydroxyalkanoate synthesis regulator	0.001 ± 0.001	0.027 ± 0.003	0.023 ± 0.002
Swol_1727	Zn-dependent dehydrogenase	ND	0.019 ± 0.004	0.028 ± 0.005
Swol_1855	60 kDa chaperonin GROEL	0.042 ± 0.001	0.027 ± 0.004	0.014 ± 0.001
Swol_1856	10 kDa chaperonin GROES	0.049 ± 0.004	0.031 ± 0.007	0.022 ± 0.003
Swol_2030	3-hydroxybutyryl-CoA dehydrogenase	0.021 ± 0.002	0.003 ± 0.001	0.005 ± 0.001
Swol_2051	Acetyl-CoA acetyltransferase	0.035 ± 0.001	0.010 ± 0.001	0.010 ± 0.0005
Swol_2148	Branched-chain amino acid aminotransferase	0.004 ± 0.001	0.006 ± 0.001	0.009 ± 0.002
Swol_2296	Hypothetical protein	0.005 ± 0.001	0.012 ± 0.001	0.010 ± 0.001
Swol_2382	Sodium-transporting two-sector ATPase	0.009 ± 0.0004	0.012 ± 0.002	0.008 ± 0.001
Swol_2386	F_0_F_1_-type ATP synthase subunit B	0.012 ± 0.001	0.010 ± 0.0004	0.009 ± 0.002

*Abbreviation: ND, not detected*.

### Beta-oxidation enzymes

**Figure 3:**
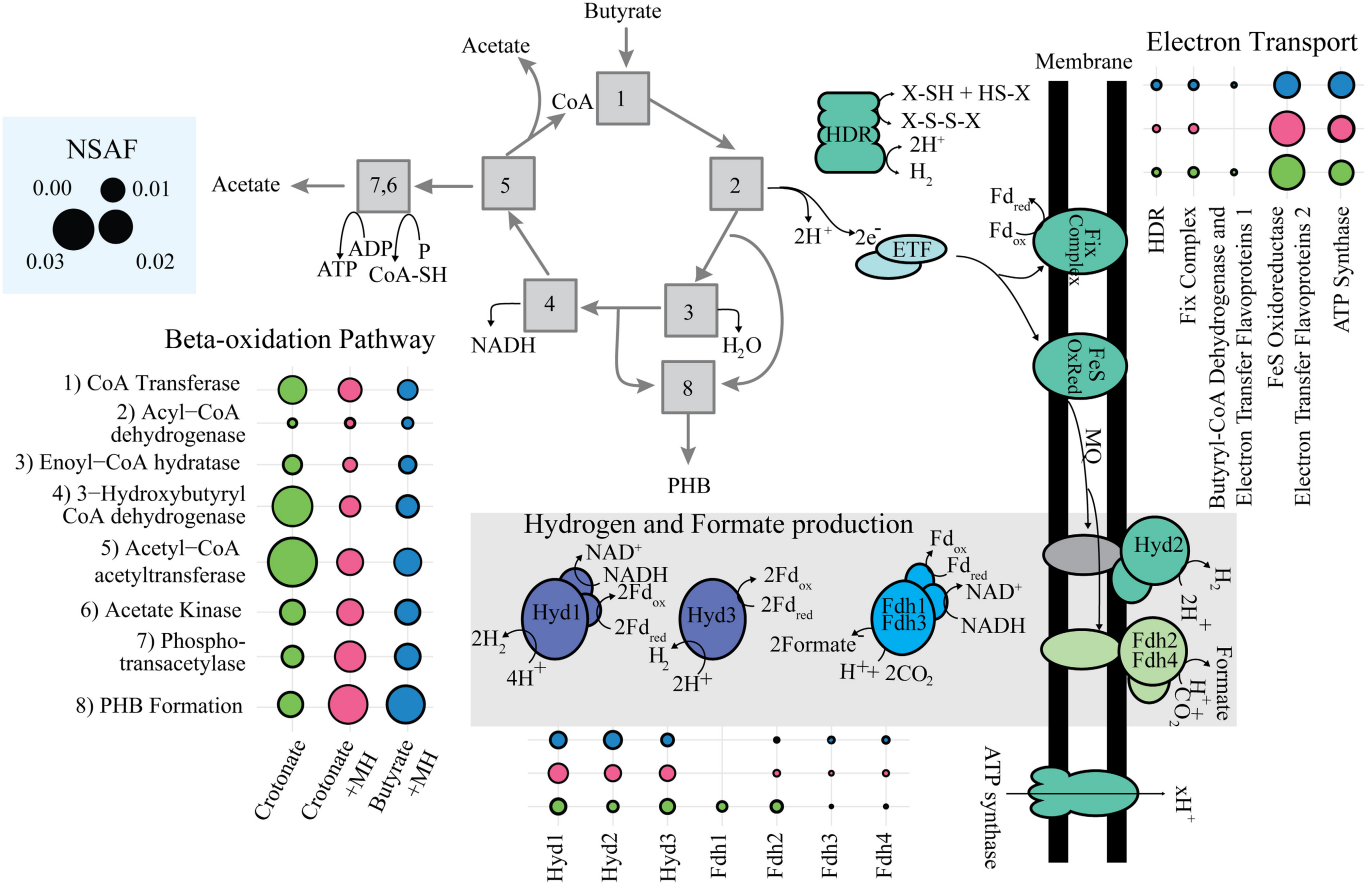
**Abundance of key enzymes of *S. wolfei*'s metabolism**. Abundance represented in the sum of NSAF for enzymes in each category. Symbols: green circle, *S. wolfei* pure culture on crotonate; pink circles, *S. wolfei-M. hungatei* coculture on crotonate; blue circles, *S. wolfei-M. hungatei* coculture on butyrate.

The abundance of beta-oxidation proteins reflect the metabolic specialization of *S. wolfei* as a bacterium that metabolizes short-chain, saturated and unsaturated fatty acids (Figure 3) (McInerney et al., 1979). Genomic analysis showed that *S. wolfei*'s genome contained multiple paralogs for each step in beta-oxidation (Sieber et al., 2010) and our whole-cell proteome analysis showed that *S. wolfei* expressed and translated multiple, paralogous, beta-oxidation enzymes. Seven acyl-CoA dehydrogenases were detected in the proteome (Figure 3; Supplemental Data Set 2). The Swol_2052 gene product was the most abundant acyl-CoA dehydrogenase and was detected in all growth conditions. Swol_2052 and Swol_1933 gene products were detected in the dominant Bcd activity purified from butyrate-grown *S. wolfei* cells (Müller et al., 2009) and subsequent proteomic analysis detected these two Bcds in crotonate-grown pure cultures and butyrate-grown cocultures of *S. wolfei* (Schmidt et al., 2013). Multiple CoA transferases, enoyl-CoA dehydratases, 3-hydroxyacyl-CoA dehydrogenases and acetyl-CoA acetyltransferases, whose abundance varied with growth condition, also were detected (Figure 3; Supplemental Data Set 2). Interestingly, gene products corresponding to a set of adjacent beta-oxidation genes and an acetate kinase (Swol_1483-1486) were not detected under any growth condition. A 3-hydroxybutyryl-CoA dehydrogenase (Swol_2030 gene product) was among the more abundant proteins detected, suggesting a specific role in oxidation/reduction when crotonate is metabolized in the absence of a suitable partner.

### Interspecies electron transfer proteins

Interspecies electron transfer is necessary for degradation of butyrate (Schink, 1997), and depending on the growth condition, *S. wolfei* can utilize either hydrogen (Sieber et al., 2014) or formate (Schmidt et al., 2013). The *S. wolfei* genome contains three hydrogenases and five formate dehydrogenases (Sieber et al., 2010). All three hydrogenases were detected in all growth conditions (Figure 3; Supplemental Data Set 2), suggesting the reoxidation of reduced electron carriers (NADH, reduced flavoproteins, and reduced ferredoxin) may involve different enzyme systems. The detected hydrogenases include the electron confurcating hydrogenase, Hyd1, which is predicted to use NADH and ferredoxin; a ferredoxin-dependent hydrogenase (Hyd3); and a membrane-bound hydrogenase (Hyd2), which may interact with the quinone pool. In contrast, the abundance of the four detected formate dehydrogenases was much lower than that of the hydrogenases (Figure 3; Supplemental Data Set 2). Hydrogenases were abundant in this study, but formate dehydrogenases were abundant when *S. wolfei* was grown under different growth conditions (Schmidt et al., 2013; Sieber et al., 2014), suggesting that relative importance of interspecies hydrogen vs. formate transfer depends on growth condition.

### Proteins necessary for reverse electron transfer

Swol_0697 and Swol_0696 gene products, which comprise the Etf complex EtfAB2, were among the most abundant proteins in the proteome in all growth conditions (Table 1; Figure 3), consistent with previous proteomic work (Schmidt et al., 2013). Adjacent to Swol_0696 and Swol_0697 is a gene for a membrane-bound FeS oxidoreductase (Swol_0698), which is postulated to be an EtfAB:menaquinone oxidoreductase (Sieber et al., 2012). The Swol_0698 gene product was highly abundant in under all growth conditions (Figure 3, Schmidt et al., 2013). This protein was present in highly purified, Bcd preparations from butyrate-grown, *S. wolfei* cells (Müller et al., 2009; Schmidt et al., 2013). Swol_0696-Swol_0698 gene products likely form a complex that functions to reduce menaquinone with electrons derived from acyl-CoA intermediates when *S. wolfei* grows on butyrate.

Proteomic analysis detected additional protein systems that could function in reverse electron transfer but these were less abundant than Swol_0696-Swol_0698 gene products (Figure 3). The Fix complex consists of FixAB, which is the EtfAB3 complex (Swol_2121 and Swol_2122 gene products), FixX, a ferredoxin (Swol_2123 gene product) and FixC, an Etf:quinone oxidoreductase (Swol_2124 gene product). The Fix proteins were ten-fold less abundant than the Swol_0696-Swol_0698 gene products under all growth conditions, suggesting that Fix may not function as a major catabolic system. However, it could function to supply reduced ferredoxin for biosynthetic processes, e.g., pyruvate synthesis from acetyl-CoA and CO_2_ or hydrogen or formate production by confurcating hydrogenases and formate dehydrogenases (Figure 3). A heterodisulfide reductase (Hdr) was also detected in the proteome. In hydrogenotrophic methanogens, this enzyme couples the unfavorable reduction of ferredoxin with electrons from hydrogen or formate to the favorable reduction of CoM-S-S-CoB heterodisulfide with electrons derived from hydrogen or formate (Costa et al., 2010; Kaster et al., 2011). We also detected proteins (Swol_0400 and Swol_402 gene products) encoded by the genes adjacent to *hdrABC*. The Swol_0402 gene product annotates as a FAD and NAD^+^-binding oxidoreductase while the Swol_0400 gene product annotates as an iron-sulfur protein. Lastly, Swol_0266-Swol_0268 gene products could function as an electron bifurcating BcdEtfAB1 complex to produce reduced ferredoxin from crotonyl-CoA and NADH (Li et al., 2008).

Key proteins needed for ATP synthesis were also abundant in all growth conditions (Figure 3; Table 1). Given that *S. wolfei* lacks respiratory systems to create a proton motive force (Sieber et al., 2012), we suggest that the ATP synthase (Swol_2381-Swol_2388) most likely functions to hydrolyze ATP to create the proton motive force.

### Identification of proteins exclusive to interspecies interactions

We identified 15 *S. wolfei* proteins unique to interspecies interactions with *M. hungatei* (Supplemental Table 2; Supplemental Figure 2). The genes for these proteins are distributed throughout the chromosome and are not co-localized or within a genomic island. A putative zinc-dependent dehydrogenase (Swol_1727 gene product) was among the most abundant proteins detected (NSAF > 0.02) when *S*. *wolfei* was grown with *M. hungatei* on either crotonate or butyrate (Table 1; Figure 2). The high abundance of this gene product is surprising because *S. wolfei* is not known to degrade or produce alcohols (McInerney et al., 1979). Interestingly, Swol_1727 contains a GroES chaperonin domain and its deduced amino acid sequence is very similar (BLAST E-value of 5e-104) to that of SYN_01269 found in another syntrophic metabolizer, *Syntrophus aciditrophicus* (McInerney et al., 2007). Swol_1727 orthologs are also found in other sequenced syntrophic metabolizers, regardless of phylogenetic lineage, including *Syntrophobacter fumaroxidans, Pelotomaculum thermopropionicum, Syntrophothermus lipocalidus*, and *Syntrophobotulus glycolicus*. The abundance of the Swol_1727 gene product in the proteome of *S. wolfei* when grown with *M. hungatei* and the occurrence of closely related genes in genomes of organisms known to be capable of syntrophic metabolism suggests that it has an important function in syntrophy.

Analysis of the other 14 proteins exclusive to interspecies interactions did not reveal any feature suggestive of unique interspecies interactions (Supplemental Table 2). Two proteins have annotated functions in beta-oxidation (Swol_1935 and Swol_1936 gene products) and one has an annotated function in poly-(3-hydroxyalkanoate) synthesis [poly-(3-hydroxyalkanoic acid) synthase, Swol_1241 gene product]. Two proteins with unknown function (Swol_1036 and Swol_2364 gene products) were detected. Swol_1036 has a nucleotidyltransferase domain and Swol_2364 has a nucleoside triphosphate pyrophosphohydrolase domain, suggesting housekeeping functions. Other proteins detected have predicted functions in cell biosynthesis (Swol_0643, Swol_0965, Swol_0975, Swol_1727, Swol_1958, and Swol_1851 gene products), energy production (Swol_1030 gene product) and replication (Swol_0001 and Swol_0002 gene products).

Eighty-three *S. wolfei* proteins were unique to the syntrophic growth on butyrate where the activity of *M. hungatei* is obligatory (Supplemental Figure 2; Supplemental Table 3). The function of 33 of these proteins is unknown (Supplemental Table 3). Of the proteins detected only in butyrate-grown *S. wolfei, four* are encoded by genes with CRISPR-associated functions [Swol_2519 (CRISPR-associated protein, Cas5), Swol_2524 (DUF324 domain-containing protein), Swol_2525 (unknown function) and Swol_2529 (DUF1887 domain-containing protein)] and their genes are located with other CRISPR-associated genes and an adjacent intergenic CRISPR region. The detection of other CRISPR-associated proteins (Swol_2520 and Swol_2522 gene products) in pure culture *S. wolfei* cells grown on crotonate shows that CRISPR function is not unique to syntrophic growth.

Seven putative transcriptional regulatory proteins were detected only in butyrate-grown *S. wolfei* cells. The function for many of these is unknown, but they likely serve important roles in modulating the physiological responses of *S. wolfei* required for syntrophic growth. The Swol_1040 gene product is signal transduction histidine kinase that contains domains similar to those of an Fe-only hydrogenase and a ferredoxin. The Swol_1040 may be part of a two-component regulator involved in the regulation of hydrogen production. Swol_0456 gene product is one of three paralogous proteins in *S. wolfei* that has PAS, sigma-54 and DNA-binding domains. PAS domains function as signal input modules in proteins that sense environmental stimuli by detecting changes in the electron transport system (Taylor and Zhulin, 1999). The Swol_1645 gene product is a redox sensitive transcriptional regulator, which in other organisms modulates transcription in response to shifts in the NADH/NAD^+^ ratio (Brekasis and Paget, 2003; Gyan et al., 2006). Five receiver only domain proteins were also identified. The detection of these regulatory proteins suggests the importance of sensing environmental and physiological signals during interspecies interactions.

Other proteins detected only in butyrate-grown cells included proteins involved in amino acid metabolism and transport, lipid metabolism and transport, nucleotide metabolism and transport and cofactor transport and metabolism (Supplemental Table 3). The number of peptides assigned to proteins involved in amino acid metabolism and transport based on COG functional classification was higher in butyrate-grown, coculture *S. wolfei* cells than crotonate-grown, pure culture *S. wolfei* cells (Supplemental Figure 3). The importance of biosynthetic capability in slow growing syntrophic coculture was unexpected. Interestingly the up-regulation of genes involved in amino acid synthesis has been detected in cocultures of termite gut spirochetes (Rosenthal et al., 2011) and in syntrophically grown *P. thermopropionicum* (Kato et al., 2009) by transcriptional analysis.

Previous work has shown that PHA production and utilization is an important intracellular process in *S. wolfei* (Amos and McInerney, 1989). This conclusion is supported by protein abundance patterns reported here. PhaR (Swol_1244 gene product), poly-(3-hydroxyalkanoic acid) synthase (Swol_1241 gene product) and an acyl-CoA dehydratase (Swol_1242 gene product) were more abundant during growth with *M. hungatei*. PHA-associated enzymes, enoyl-CoA hydratases (Swol_0487 gene product) and acetoacetyl-CoA reductase (Swol_0651 gene product), were detected only in butyrate-grown cells (Supplemental Table 3).

Nine proteins unique to *S. wolfei-M. hungatei* coculture growth on crotonate were detected (Supplemental Table 4). Here, the presence of *M. hungatei* is not obligatory for crotonate metabolism by *S. wolfei*. The proteins found exclusively during coculture growth on crotonate include a transcriptional regulator with a HD-GYP domain, which may function as a phosphodiesterase to control cyclic nucleotide levels (Marinez et al., 2002), a putative NAD(P)H-flavin oxidoreductase function (Swol_1523 gene product), and three proteins with unknown functions (Supplemental Table 3).

This extensive proteomic analysis defines the physiological response of *S. wolfei* to the syntrophic lifestyle. NMDS analysis showed that *S. wolfei* adjusted its physiology in response to the methanogen. An uncharacterized, membrane-bound iron-sulfur oxidoreductase and EtfAB2 were abundant under all growth conditions and may provide the conduit for electron transfer between Bcd and the menaquinone pool. Reoxidation of menaquinol by a membrane-bound hydrogenase (Hyd2) provides a mechanism for the reverse electron transfer of electrons derived from butyryl-CoA oxidation to hydrogen using the proton motive force (Figure 3). Hydrogenases were abundant in this study, but formate dehydrogenases were abundant when *S. wolfei* is grown under different growth conditions (Schmidt et al., 2013; Sieber et al., 2014), suggesting that relative importance of interspecies hydrogen vs. formate transfer depends on growth condition. A GroES domain-containing, zinc-dependent dehydrogenase (Swol_1727 gene product) and several transcriptional regulators, responsive to environmental stimuli or cellular physiological status, were detected when *S. wolfei* was grown the *M. hungatei*. Overall, the proteomic analysis revealed an emphasis energy metabolism and macromolecular stability by the metabolic specialist, *S. wolfei*, and the involvement of regulatory proteins responsive to environmental and physiological signals during interspecies interactions.

### Conflict of interest statement

The authors declare that the research was conducted in the absence of any commercial or financial relationships that could be construed as a potential conflict of interest.

## References

[B1] Amos D. A. McInerney M. J. (1989). Poly-β-hydroxyalkanoate in *Syntrophomonas wolfei*. Arch. Microbiol. 152, 172–177 10.1007/BF00456097

[B2] Amos D. A. McInerney M. J. (1990). Growth of *Syntrophomonas wolfei* on unsaturated short chain fatty acids. Arch. Microbiol. 154, 31–36 10.1007/BF00249174

[B3] Balch W. E. Wolfe R. S. (1976). New approach to the cultivation of methanogenic bacteria: 2-mercaptoethanesulfonic acid (HS-CoM)-dependent growth of *Methanobacterium ruminantium* in a pressurized atmosphere. Appl. Environ. Microbiol. 32, 781–791. 827241 10.1128/aem.32.6.781-791.1976 PMC170461

[B4] Beaty P. S. McInerney M. J. (1987). Growth of *Syntrophomonas wolfei* in pure culture on crotonate. Arch. Microbiol. 147, 389–393 10.1007/BF00406138

[B5] Brekasis D. Paget M. S. (2003). A novel sensor of NADH/NAD^+^ redox poise in *Streptomyces coelicolor* A3(2). EMBO J. 22, 4856–4865. 10.1093/emboj/cdg453 12970197 PMC212721

[B6] Costa K. C. Wong P. M. Wang T. Lie T. J. Dodsworth J. A. Swanson I.. (2010). Protein complexing in a methanogen suggests electron bifurcation and electron delivery from formate to heterodisulfide reductase. Proc. Nat. Acad. Sci. U.S.A. 107, 11050–11055. 10.1073/pnas.1003653107 20534465 PMC2890747

[B7] Elias J. Gygi S. (2007). Target-decoy search strategy for increased confidence in large-scale protein identifications by mass spectrometry. Nat. Methods 4, 207–214. 10.1038/nmeth1019 17327847

[B8] Eng J. K. Mccormack A. L. Yates J. R. Iii (1994). An approach to correlate tandem mass spectral data of peptides with amino acid sequences in a protein database. J. Am. Soc. Mass Spectrom. 5, 976–989. 10.1016/1044-0305(94)80016-2 24226387

[B9] Gyan S. Shiohira Y. Sato I. Takeuchi M. Sato T. (2006). Regulatory loop between redox sensing of the NADH/NAD^+^ ratio by Rex (YdiH) and oxidation of NADH by NADH dehydrogenase Ndh in *Bacillus subtilis*. J. Bacteriol. 188, 7062–7071. 10.1128/JB.00601-06 17015645 PMC1636230

[B10] Hervey W. J. Khalsa-Moyers G. Lankford P. K. Owens E. T. Mckeown C. K. Lu T. Y.. (2009). Evaluation of affinity-tagged protein expression strategies using local and global isotope ratio measurements. J. Proteome Res. 8, 3675–3688. 10.1021/pr801088f 19459691

[B11] Kaster A.-K. Moll J. Parey K. Thauer R. K. (2011). Coupling of ferredoxin and heterodisulfide reduction via electron bifurcation in hydrogenotrophic methanogenic archaea. Proc. Nat. Acad. Sci. U.S.A. 108, 2981–2986. 10.1073/pnas.1016761108 21262829 PMC3041090

[B12] Kato S. Kosaka T. Watanabe K. (2009). Substrate dependent transcriptomic shifts in *Pelotomaculum thermopropionicum* grown in syntrophic co culture with *Methanothermobacter thermautotrophicus*. Microb. Biotechnol. 2, 575–584. 10.1111/j.1751-7915.2009.00102.x 21255290 PMC3815365

[B13] Li F. Hinderberger J. Seedorf H. Zhang J. Buckel W. Thauer R. K. (2008). Coupled ferredoxin and crotonyl-coenzyme A (CoA) reduction with NADH catalyzed by the butyryl-CoA dehydrogenase/Etf complex from *Clostridium kluyveri*. J. Bacteriol. 190, 843–850. 10.1128/JB.01417-07 17993531 PMC2223550

[B14] Lu P. Vogel C. Wang R. Yao X. Marcotte E. M. (2007). Absolute protein expression profiling estimates the relative contributions of transcriptional and translational regulation. Nature Biotechnol. 25, 117–124. 10.1038/nbt1270 17187058

[B15] Mancuso F. Bunkenborg J. Wierer M. Molina H. (2012). Data extraction from proteomics raw data: an evaluation of nine tandem MS tools using Orbitrap data set. J. Proteom. 75, 5293–5303. 10.1016/j.jprot.2012.06.012 22728601

[B16] Marinez S. E. Beavo J. A. Hol W. G. J. (2002). GAF domains: two-billion-year-old molecular switches that bind cyclic nucleotides. Mol. Interv. 2, 317–323. 10.1124/mi.2.5.317 14993386

[B17] McInerney M. J. Bryant M. P. Hespell R. B. Costerton J. W. (1981). *Syntrophomonas wolfei* gen. nov. sp. nov., an anaerobic, syntrophic, fatty acid-oxidizing bacterium. Appl. Environ. Microbiol. 41, 1029–1039. 16345745 10.1128/aem.41.4.1029-1039.1981 PMC243852

[B18] McInerney M. J. Bryant M. P. Pfennig N. (1979). Anaerobic bacterium that degrades fatty acids in syntrophic association with methanogens. Arch. Microbiol. 122, 129–135 10.1007/BF00411351 9914307

[B19] McInerney M. J. Rohlin L. Mouttaki H. Kim U. Krupp R. S. Rios-Hernandez L.. (2007). The genome of *Syntrophus aciditrophicus*: life at the thermodynamic limit of microbial growth. Proc. Nat. Acad. Sci. U.S.A. 104, 7600–7605. 10.1073/pnas.0610456104 17442750 PMC1863511

[B20] McInerney M. J. Struchtemeyer C. G. Sieber J. Mouttaki H. Stams A. J. M. Schink B.. (2008). Physiology, ecology, phylogeny, and genomics of microorganisms capable of syntrophic metabolism. Ann. N.Y. Acad. Sci. 1125, 58–72. 10.1196/annals.1419.005 18378587

[B21] Meyer B. Kuehl J. Deutschbauer A. M. Price M. N. Arkin A. P. Stahl D. A. (2013a). Variation among *Desulfovibrio* apecies in electron transfer systems used for syntrophic growth. J. Bacteriol. 195, 990–1004. 10.1128/JB.01959-12 23264581 PMC3571329

[B22] Meyer B. Kuehl J. V. Deutschbauer A. M. Arkin A. P. Stahl D. A. (2013b). Flexibility of syntrophic enzyme systems in *Desulfovibrio* species ensures their adaptation capability to environmental changes. J. Bacteriol. 195, 4900–4914. 10.1128/JB.00504-13 23974031 PMC3807489

[B23] Moore R. Young M. Lee T. (2002). Qscore: an algorithm for evaluating SEQUEST database search results. J. Am. Soc. Mass Spectrom. 13, 378–386. 10.1016/S1044-0305(02)00352-5 11951976

[B24] Müller N. Schleheck D. Schink B. (2009). Involvement of NADH: acceptor oxidoreductase and butyryl-CoA dehydrogenase in reversed electron transport during syntrophic butyrate oxidation by *Syntrophomonas wolfei*. J. Bacteriol. 191, 6167–6177. 10.1128/JB.01605-08 19648244 PMC2747885

[B25] Oksanen J. Blanchet F. G. Kindt R. Legendre P. O'Hara R. B. Simpson G. L. (2011). VEGAN: Community Ecology Package. R package version 1.17-6 ed.

[B26] R Development Core Team. (2011). R: A Language and Environment for Statistical Computing (Vienna: R Foundation for Statistical Computing).

[B27] Rosenthal A. Z. Matson E. G. Eldar A. Leadbetter J. R. (2011). RNA-seq reveals cooperative metabolic interactions between two termite-gut spirochete species in co-culture. ISME J. 5, 1133–1142. 10.1038/ismej.2011.3 21326336 PMC3146290

[B28] Sato K. Nishina Y. Setoyama C. Miura R. Shiga K. (1999). Unusually high standard redox potential of acrylyl-CoA/propionyl-CoA couple among enoyl-CoA/acyl-CoA couples: a reason for the distinct metabolic pathway of propionyl-CoA from longer acyl-CoAs. J. Biochem. 126, 668–675. 10.1093/oxfordjournals.jbchem.a022501 10502673

[B29] Schink B. (1997). Energetics of syntrophic cooperation in methanogenic degradation. Microbiol. Mol. Biol. Rev. 61, 262–280. 9184013 10.1128/mmbr.61.2.262-280.1997 PMC232610

[B30] Schink B. Stams A. J. M. (2006). Syntrophism among prokaryotes, in The Prokaryotes: An Evolving Electronic Resource for the Microbiological Community, 3rd Edn., eds Dworkin M. Falkow S. Rosenberg E. Schleifer K. H. Stackebrandt E. (New York, NY: Springer-Verlag), 309–335.

[B31] Schmidt A. Müller N. Schink B. Schleheck D. (2013). A proteomic view at the biochemistry of syntrophic butyrate oxidation in *Syntrophomonas wolfei*. PLoS ONE 8:e56905. 10.1371/journaone.0056905 23468890 PMC3582634

[B32] Sieber J. R. Le H. M. McInerney M. J. (2014). The importance of hydrogen and formate transfer for syntrophic fatty, aromatic and alicyclic metabolism. Environ. Microbiol. 16, 177–188. 10.1111/1462-2920.12269 24387041

[B33] Sieber J. R. McInerney M. J. Gunsalus R. P. (2012). Genomic insights into syntrophy: the paradigm for anaerobic metabolic cooperation. Ann. Rev. Microbiol. 66, 429–452. 10.1146/annurev-micro-090110-102844 22803797

[B34] Sieber J. R. Sims D. R. Han C. Kim E. Lykidis A. Lapidus A. L.. (2010). The genome of *Syntrophomonas wolfei*: new insights into syntrophic metabolism and biohydrogen production. Environ. Microbiol. 12, 2289–2301. 10.1111/j.1462-2920.2010.02237.x 21966920

[B35] Stams A. J. Plugge C. M. (2009). Electron transfer in syntrophic communities of anaerobic bacteria and archaea. Nat. Rev. Microbiol. 7, 568–577. 10.1038/nrmicro2166 19609258

[B36] Tabb D. L. Mcdonald W. H. Yates J. R. III. (2002). DTASelect and contrast: tools for assembling and comparing protein identifications from shotgun proteomics. J. Proteome Res. 1, 21–26. 10.1021/pr015504q 12643522 PMC2811961

[B37] Taylor B. L. Zhulin I. B. (1999). PAS domains: internal sensors of oxygen, redox potential, and light. Microbiol. Mol. Biol. Rev. 63, 479–506. 10357859 10.1128/mmbr.63.2.479-506.1999 PMC98974

[B38] Thompson M. R. Chourey K. Froelich J. M. Erickson B. K. Verberkmoes N. C. Hettich R. L. (2008). Experimental approach for deep proteome measurements from small-scale microbial biomass samples. Anal. Chem. 80, 9517–9525. 10.1021/ac801707s 19072265

[B39] Wallrabenstein C. Schink B. (1994). Evidence of reversed electron-transport in syntrophic butyrate or benzoate oxidation by *Syntrophomonas wolfei* and *Syntrophus buswellii*. Arch. Microbiol. 162, 136–142 10.1007/BF00264387

[B40] Washburn M. P. Wolters D. Yates J. R. III. (2001). Large-scale analysis of the yeast proteome by multidimensional protein identification technology. Nat. Biotechnol. 19, 242–247. 10.1038/85686 11231557

[B41] Wolters D. A. Washburn M. P. Yates J. R. III. (2001). An automated multidimensional protein identification technology for shotgun proteomics. Anal. Chem. 73, 5683–5690. 10.1021/ac010617e 11774908

[B42] Zybailov B. Mosley A. L. Sardiu M. E. Coleman M. K. Florens L. Washburn M. P. (2006). Statistical analysis of membrane proteome expression changes in *Saccharomyces cerevisiae*. J. Proteome Res. 5, 2339–2347. 10.1021/pr060161n 16944946

