# Supplementary material for: Proteomic analysis reveals metabolic and regulatory systems involved in the syntrophic and axenic lifestyle of Syntrophomonas wolfei
Source: Front Microbiol. 2015 Feb 11;6:115. doi: 10.3389/fmicb.2015.00115 (PMC4324140; doi:10.3389/fmicb.2015.00115)
Supplement: Supplementary file 1 [file SupplementalMaterials.DOCX]

***Supplementary Material***

**Proteomic analysis reveals metabolic and regulatory systems involved the syntrophic and axenic lifestyle of *Syntrophomonas wolfei*.**

**Jessica R. Sieber^1^, Bryan R. Crable^1^, Cody S. Sheik^2^, Gregory B. Hurst^3^, Lars Rohlin^4^, Robert P. Gunsalus^4^ and Michael J. McInerney^1^***

^1^Department of Botany and Microbiology, University of Oklahoma
^2^Department of Geological Sciences, University of Michigan
^3^Oak Ridge National Laboratory
^4^Department of Microbiology, Immunology, and Molecular Genetics, University of California, Los Angeles, CA 90095-1489, USA.

*** Correspondence: Michael J. McInerney**, ^1^Department of Botany and Microbiology, University of Oklahoma. 770 Van Vleet Oval, Norman, OK, 73019, USA.
mcinerney@ou.edu

1. **Supplementary Data**

**Supplemental Data Sheet 1. Abundance of all detected *S. wolfei* proteins under axenic and syntrophic growth.**

**Supplemental Data Sheet 2. Sum of NSAF values for each enzyme system shown in Figure 3 grouped by function.**

1. **Supplementary Figures and Tables**

## Supplementary Tables

**Supplemental Table 1. Constitutively abundant proteins during axenic and syntrophic growth of *S. wolfei.***

| Locus Tag | Crotonate | Crotonate with *M.hungatei* | Butyrate with *M.hungatei* | Predicted Function |
| --- | --- | --- | --- | --- |
| Swol_0009 | 0.00127 | 0.00163 | 0.00189 | Phosphoglycerate dehydrogenase |
| Swol_0047 | 0.01055 | 0.01178 | 0.01304 | transcription regulator AbrB |
| Swol_0069 | 0.00272 | 0.00220 | 0.00314 | ribosomal protein L25 |
| Swol_0095 | 0.00048 | 0.00036 | 0.00066 | ATP-dependent metalloprotease FtsH |
| Swol_0118 | 0.00077 | 0.00097 | 0.00077 | hypothetical protein |
| Swol_0141 | 0.00270 | 0.00361 | 0.00372 | hypothetical protein |
| Swol_0272 | 0.00036 | 0.00024 | 0.00034 | Glyceraldehyde-3-phosphate dehydrogenase |
| Swol_0273 | 0.00042 | 0.00024 | 0.00028 | Phosphoglycerate kinase |
| Swol_0316 | 0.00480 | 0.00519 | 0.00487 | extracellular solute-binding protein, family 3 |
| Swol_0331 | 0.00469 | 0.00458 | 0.00524 | extracellular solute-binding protein, family 7 |
| Swol_0362 | 0.00071 | 0.00073 | 0.00077 | Tryptophan synthase |
| Swol_0374 | 0.00033 | 0.00032 | 0.00033 | aspartyl/glutamyl-tRNA amidotransferase subunit B |
| Swol_0376 | 0.00110 | 0.00120 | 0.00109 | 3-isopropylmalate dehydratase |
| Swol_0436 | 0.01162 | 0.00802 | 0.00589 | 4-hydroxybutyrate coenzyme A transferase |
| Swol_0443 | 0.00032 | 0.00043 | 0.00033 | Coproporphyrinogen III oxidase |
| Swol_0445 | 0.00094 | 0.00099 | 0.00067 | Magnesium chelatase |
| Swol_0446 | 0.00030 | 0.00037 | 0.00043 | Mg-chelatase subunit ChlD-like protein |
| Swol_0459 | 0.00124 | 0.00108 | 0.00164 | hypothetical protein |
| Swol_0462 | 0.00357 | 0.00472 | 0.00506 | thioredoxin |
| Swol_0468 | 0.00050 | 0.00057 | 0.00065 | Alanine--tRNA ligase |
| Swol_0490 | 0.00192 | 0.00173 | 0.00205 | chaperone protein DnaK |
| Swol_0493 | 0.00074 | 0.00097 | 0.00083 | putative ATPase with chaperone activity |
| Swol_0585 | 0.00078 | 0.00074 | 0.00053 | arginine repressor |
| Swol_0588 | 0.01147 | 0.01562 | 0.01288 | heat shock protein, molecular chaperone |
| Swol_0590 | 0.00038 | 0.00047 | 0.00035 | response regulator receiver protein |
| Swol_0641 | 0.00039 | 0.00029 | 0.00049 | Thiamine-phosphate diphosphorylase |
| Swol_0651 | 0.00050 | 0.00042 | 0.00044 | short chain dehydrogenase/reductase family |
| Swol_0655 | 0.00397 | 0.00458 | 0.00326 | hypothetical protein |
| Swol_0659 | 0.00060 | 0.00048 | 0.00040 | PpiC-type peptidyl-prolyl cis-trans isomerase |
| Swol_0665 | 0.00088 | 0.00061 | 0.00076 | hypothetical protein |
| Swol_0672 | 0.00061 | 0.00067 | 0.00048 | Tryptophan--tRNA ligase |
| Swol_0675 | 0.00436 | 0.00229 | 0.00318 | Acetyl-CoA C-acetyltransferase |
| Swol_0688 | 0.00132 | 0.00100 | 0.00112 | metallo cofactor biosynthesis protein |
| Swol_0691 | 0.00026 | 0.00037 | 0.00031 | radical SAM domain protein |
| Swol_0698 | 0.00514 | 0.00456 | 0.00523 | putative iron-sulfur-binding reductase |
| Swol_0767 | 0.00894 | 0.01014 | 0.00904 | phosphate acetyltransferase |
| Swol_0771 | 0.00042 | 0.00045 | 0.00053 | hypothetical protein |
| Swol_0808 | 0.00141 | 0.00126 | 0.00115 | beta-lactamase-like protein |
| Swol_0833 | 0.00112 | 0.00091 | 0.00154 | hypothetical protein |
| Swol_0840 | 0.00102 | 0.00081 | 0.00067 | DNA topoisomerase |
| Swol_0844 | 0.00032 | 0.00036 | 0.00029 | heat shock protein HslVU, ATPase subunit |
| Swol_0874 | 0.00022 | 0.00028 | 0.00024 | CheA signal transduction histidine kinases |
| Swol_0883 | 0.00374 | 0.00455 | 0.00317 | elongation factor Ts |
| Swol_0884 | 0.00080 | 0.00041 | 0.00043 | hypothetical protein |
| Swol_0919 | 0.00107 | 0.00103 | 0.00158 | coproporphyrinogen III oxidase |
| Swol_0927 | 0.00088 | 0.00053 | 0.00079 | methyl-accepting chemotaxis sensory transducer |
| Swol_0942 | 0.00023 | 0.00031 | 0.00032 | S-layer domain-like protein |
| Swol_0973 | 0.00135 | 0.00084 | 0.00073 | hypothetical protein |
| Swol_1013 | 0.00042 | 0.00040 | 0.00059 | D-lactate dehydrogenase |
| Swol_1017 | 0.00430 | 0.00329 | 0.00292 | iron hydrogenase, small subunit |
| Swol_1019 | 0.00413 | 0.00242 | 0.00221 | Fe-hydrogenase, gamma subunit |
| Swol_1090 | 0.00137 | 0.00146 | 0.00150 | hypothetical protein |
| Swol_1110 | 0.00083 | 0.00084 | 0.00121 | Tyrosine--tRNA ligase |
| Swol_1152 | 0.00286 | 0.00401 | 0.00314 | translation elongation factor G |
| Swol_1155 | 0.00062 | 0.00042 | 0.00064 | D-alanine--D-alanine ligase |
| Swol_1234 | 0.00205 | 0.00239 | 0.00223 | Methionine adenosyltransferase |
| Swol_1250 | 0.00221 | 0.00153 | 0.00290 | stage V sporulation protein S |
| Swol_1254 | 0.00076 | 0.00080 | 0.00073 | HDIG/KH domain protein |
| Swol_1255 | 0.00217 | 0.00263 | 0.00325 | hypothetical protein |
| Swol_1269 | 0.00065 | 0.00051 | 0.00078 | Dihydrodipicolinate synthase |
| Swol_1270 | 0.00147 | 0.00112 | 0.00115 | aspartate-semialdehyde dehydrogenase |
| Swol_1344 | 0.00170 | 0.00196 | 0.00143 | hydroxymethylbutenyl pyrophosphate reductase |
| Swol_1347 | 0.00082 | 0.00081 | 0.00073 | 3-phosphoshikimate 1-carboxyvinyltransferase |
| Swol_1373 | 0.00033 | 0.00029 | 0.00027 | Precorrin-6B methylase 2-like protein |
| Swol_1409 | 0.00063 | 0.00062 | 0.00074 | Formate--tetrahydrofolate ligase |
| Swol_1431 | 0.00063 | 0.00068 | 0.00075 | Holliday junction DNA helicase RuvB |
| Swol_1497 | 0.00193 | 0.00257 | 0.00225 | 30S ribosomal protein S16 |
| Swol_1514 | 0.00066 | 0.00048 | 0.00065 | CBS domain protein |
| Swol_1536 | 0.00053 | 0.00080 | 0.00070 | hypothetical protein |
| Swol_1565 | 0.00014 | 0.00021 | 0.00014 | metal dependent phosphohydrolase |
| Swol_1577 | 0.00066 | 0.00041 | 0.00037 | chaperone DnaJ |
| Swol_1578 | 0.00460 | 0.00333 | 0.00322 | chaperone DnaK |
| Swol_1579 | 0.00451 | 0.00493 | 0.00552 | GrpE protein |
| Swol_1605 | 0.00214 | 0.00187 | 0.00257 | O-acetylserine (thiol)-lyase |
| Swol_1607 | 0.00087 | 0.00117 | 0.00111 | Glutamate-5-semialdehyde dehydrogenase |
| Swol_1611 | 0.00330 | 0.00291 | 0.00235 | Ribosomal protein L27 |
| Swol_1613 | 0.00160 | 0.00122 | 0.00116 | Ribosomal protein L21 |
| Swol_1641 | 0.00096 | 0.00095 | 0.00076 | cell shape determining protein, MreB/Mrl |
| Swol_1734 | 0.00047 | 0.00056 | 0.00052 | hypothetical protein |
| Swol_1784 | 0.00047 | 0.00057 | 0.00064 | GMP synthase |
| Swol_1785 | 0.00071 | 0.00066 | 0.00054 | Hypoxanthine phosphoribosyltransferase |
| Swol_1841 | 0.00056 | 0.00037 | 0.00057 | Butyryl-CoA dehydrogenase |
| Swol_2088 | 0.00287 | 0.00253 | 0.00357 | hypothetical protein |
| Swol_2113 | 0.00120 | 0.00084 | 0.00102 | branched-chain amino acid ABC transporter, periplasmic amino acid-binding protein |
| Swol_2122 | 0.00181 | 0.00167 | 0.00197 | hypothetical protein |
| Swol_2126 | 0.00113 | 0.00112 | 0.00169 | acyl-CoA dehydrogenase, short-chain |
| Swol_2145 | 0.00157 | 0.00172 | 0.00135 | acetolactate synthase, small subunit |
| Swol_2162 | 0.00033 | 0.00020 | 0.00033 | methyl-accepting chemotaxis sensory transducer |
| Swol_2167 | 0.00068 | 0.00060 | 0.00075 | hypothetical protein |
| Swol_2173 | 0.00157 | 0.00162 | 0.00202 | hypothetical protein |
| Swol_2287 | 0.00038 | 0.00055 | 0.00040 | Ornithine carbamoyltransferase |
| Swol_2297 | 0.00125 | 0.00069 | 0.00071 | ribosomal protein S9 |
| Swol_2304 | 0.00109 | 0.00075 | 0.00098 | DNA-directed RNA polymerase |
| Swol_2306 | 0.00489 | 0.00493 | 0.00473 | ribosomal protein S11 |
| Swol_2312 | 0.00172 | 0.00115 | 0.00106 | Adenylate kinase |
| Swol_2315 | 0.00235 | 0.00252 | 0.00219 | ribosomal protein L30 |
| Swol_2318 | 0.00363 | 0.00316 | 0.00359 | ribosomal protein L6 |
| Swol_2330 | 0.00174 | 0.00183 | 0.00238 | ribosomal protein L2 |
| Swol_2332 | 0.00284 | 0.00274 | 0.00198 | ribosomal protein L4/L1e |
| Swol_2333 | 0.00156 | 0.00096 | 0.00130 | 50S ribosomal protein L3 |
| Swol_2338 | 0.00197 | 0.00179 | 0.00215 | ribosomal protein S12 |
| Swol_2341 | 0.00078 | 0.00104 | 0.00093 | DNA-directed RNA polymerase |
| Swol_2343 | 0.00425 | 0.00511 | 0.00583 | ribosomal protein L7/L12 |
| Swol_2346 | 0.00196 | 0.00122 | 0.00134 | ribosomal protein L11 |
| Swol_2382 | 0.00932 | 0.01300 | 0.00892 | Sodium-transporting two-sector ATPase |
| Swol_2383 | 0.00722 | 0.00746 | 0.00821 | Sodium-transporting two-sector ATPase |
| Swol_2384 | 0.00503 | 0.00542 | 0.00502 | Sodium-transporting two-sector ATPase |
| Swol_2385 | 0.00971 | 0.00874 | 0.00870 | ATP synthase F1 delta subunit |
| Swol_2386 | 0.01190 | 0.01078 | 0.01050 | F0F1-type ATP synthase subunit b-like protein |
| Swol_2408 | 0.00120 | 0.00092 | 0.00065 | transcription termination factor Rho |
| Swol_2539 | 0.00286 | 0.00336 | 0.00311 | single-strand binding protein |
| Swol_2540 | 0.00341 | 0.00504 | 0.00458 | ribosomal protein S6 |
| Swol_2560 | 0.00034 | 0.00044 | 0.00038 | selenocysteine-specific translation elongation factor |

**Supplemental Table 2. Proteins detected only during growth with *M. hungatei.***

| Locus Tag | Crotonate with JF1 | Butyrate with JF1 | Predicted Function |
| --- | --- | --- | --- |
| Swol_1935 | 0.0061±0.0015 | 0.0036±0.0007 | 3-hydroxybutyryl-CoA dehydrogenase |
| Swol_1936 | 0.0028±0.0007 | 0.0037±0.0006 | 3-hydroxybutyryl-CoA dehydratase |
| Swol_1938 | 0.0006±0.0002 | 0.0006±0.0000 | PAS/PAC domain-like protein |
| Swol_1241 | 0.0003±0.0001 | 0.0002±0.0001 | Poly(R)-hydroxyalkanoic acid synthase, PhaC |
| Swol_1727 | 0.0190±0.0037 | 0.0281±0.0046 | Zinc-binding dehydrogenase |
| Swol_1850 | 0.0008±0.0003 | 0.0008±0.0002 | Malonyl-CoA-[acyl-carrier-protein] transacylase |
| Swol_1851 | 0.0005±0.0003 | 0.0006±0.0001 | Malonyl-CoA-[acyl-carrier-protein] transacylase |
| Swol_0643 | 0.0003±0.0001 | 0.0005±0.0000 | Thiamine biosynthesis protein thiC |
| Swol_0965 | 0.0004±0.0001 | 0.0002±0.0001 | 1,2-diacylglycerol 3-glucosyltransferase |
| Swol_0975 | 0.0005±0.0001 | 0.0004±0.0001 | Hydantoinase/oxoprolinase |
| Swol_1030 | 0.0004±0.0001 | 0.0007±0.0002 | Formate dehydrogenase accessory protein |
| Swol_0001 | 0.0002±0.0000 | 0.0003±0.0000 | Chromosomal replication initiator protein dnaA |
| Swol_0002 | 0.0003±0.0001 | 0.0008±0.0001 | DNA polymerase III, beta chain |
| Swol_1036 | 0.0003±0.0000 | 0.0003±0.0001 | Unknown function |
| Swol_2364 | 0.0018±0.0015 | 0.0007±0.0001 | Unknown function |

**Supplemental Table 3. Proteins found only during syntrophic growth on butyrate.**

| Locus Tag | NSAF | Predicted function | |
| --- | --- | --- | --- |
| Function unknown | | |  |
| Swol_0012 | 0.00021 | Unknown function | |
| Swol_0043 | 0.00023 | Unknown function | |
| Swol_0082 | 0.00024 | MazG protein |  |
| Swol_0111 | 0.00045 | Unknown function | |
| Swol_0165 | 0.00017 | WD40-like repeat protein | |
| Swol_0224 | 0.00082 | Unknown function | |
| Swol_0456 | 0.00017 | Unknown function | |
| Swol_0477 | 0.00018 | Unknown function | |
| Swol_0530 | 0.00049 | Unknown function | |
| Swol_0750 | 0.00062 | Unknown function | |
| Swol_0870 | 0.00033 | Unknown function | |
| Swol_0920 | 0.00041 | Unknown function | |
| Swol_0924 | 0.0004 | Beta-lactamase-like protein | |
| Swol_0990 | 0.00007 | Unknown function | |
| Swol_0998 | 0.00014 | Unknown function | |
| Swol_1002 | 0.00276 | Unknown function | |
| Swol_1158 | 0.00027 | Unknown function | |
| Swol_1246 | 0.00045 | Unknown function | |
| Swol_1257 | 0.00035 | Unknown function | |
| Swol_1372 | 0.00075 | Unknown function | |
| Swol_1404 | 0.00093 | Unknown function | |
| Swol_1458 | 0.00016 | Unknown function | |
| Swol_1511 | 0.00023 | Unknown function | |
| Swol_1528 | 0.0004 | Unknown function | |
| Swol_1647 | 0.00025 | Unknown function | |
| Swol_1808 | 0.00042 | Unknown function | |
| Swol_1812 | 0.00029 | Unknown function | |
| Swol_2089 | 0.00031 | Unknown function | |
| Swol_2494 | 0.00016 | Unknown function | |
| Swol_2519 | 0.00032 | Unknown function | |
| Swol_2524 | 0.00013 | Unknown function | |
| Swol_2525 | 0.00034 | Unknown function | |
| Swol_2529 | 0.00053 | Unknown function | |
| Amino acid metabolism and transport | | |  |
| Swol_0008 | 0.00015 | Serine-glyoxylate transaminase | |
| Swol_0529 | 0.00046 | 3-dehydroquinate synthase | |
| Swol_0668 | 0.00033 | Spermidine synthase | |
| Swol_0669 | 0.00023 | Agmatinase |  |
| Swol_0712 | 0.00009 | Putative asparagine synthetase | |
| Swol_0955 | 0.00025 | Pyrroline-5-carboxylate reductase | |
| Swol_1007 | 0.00026 | Glutamate-ammonia ligase | |
| Swol_1198 | 0.00037 | Histidinol-phosphate aminotransferase | |
| Swol_1249 | 0.00027 | Zn-dependent dipeptidase, microsomal dipeptidase | |
| Swol_1273 | 0.00029 | Dihydrodipicolinate reductase | |
| Swol_1302 | 0.00031 | Threonine synthase | |
| Swol_1349 | 0.0005 | Prephenate dehydrogenase | |
| Swol_1767 | 0.00044 | Imidazoleglycerol-phosphate dehydratase | |
| Swol_1769 | 0.0006 | ATP phosphoribosyltransferase | |
| Swol_1982 | 0.0006 | Glycine cleavage system H protein | |
| Swol_2542 | 0.00025 | Selenophosphate synthase | |
| Swol_2552 | 0.00026 | Branched-chain amino acid ABC transporter | |
| Coenzyme transport and metabolism | | |  |
| Swol_0694 | 0.00048 | Glutamate-1-semialdehyde-2,1-aminomutase | |
| Swol_1645 | 0.00064 | CoA-binding |  |
| Energy production and conversion | | |  |
| Swol_0519 | 0.00013 | Pyruvate carboxylase, PYKA | |
| Swol_1629 | 0.00038 | Tartrate dehydratase alpha subunit | |
| Inorganic ion transport and metabolism | | |  |
| Swol_0661 | 0.00039 | Fe^2+^ transport system protein B-like protein | |
| Swol_1005 | 0.00023 | Putative aluminum resistance protein | |
| Replication, recombination, and repair | | |  |
| Swol_0475 | 0.00013 | CRISPR-associated helicase Cas3 domain protein | |
| Swol_0838 | 0.00015 | Site-specific DNA-methyltransferase | |
| Swol_0334 | 0.00092 | Response regulator receiver protein | |
| Translation, ribosome structure and biogenesis | | |  |
| Swol_0372 | 0.00116 | Glutamyl-tRNA amidotransferase, C subunit | |
| Swol_1576 | 0.00037 | Ribosomal protein L11 methyltransferase-like protein | |
| Transcription | | |  |
| Swol_2572 | 0.00019 | GTPase |  |
| Signal transduction | | |  |
| Swol_0334 | 0.00092 | Response regulator receiver protein | |
| Swol_0337 | 0.00013 | Metal dependent phosphohydrolase | |
| Swol_1040 | 0.00011 | Signal transduction histidine kinase regulating C4-dicarboxylate transport system protein | |
| Swol_1041 | 0.00019 | Response regulator receiver protein | |
| Swol_2130 | 0.00033 | Response regulator receiver protein | |
| Swol_2131 | 0.0009 | Response regulator receiver protein | |
| Swol_2393 | 0.00076 | Protein tyrosine phosphatase | |
| Lipid transport and metabolism | | |  |
| Swol_0411 | 0.00023 | CoA enzyme activase | |
| Swol_0428 | 0.00056 | (R)-hydroxyglutaryl-CoA dehydratase activator | |
| Swol_0487 | 0.00068 | MaoC-like dehydratase | |
| Swol_0488 | 0.00053 | Butyryl-CoA dehydrogenase | |
| Swol_1480 | 0.00043 | Zn-dependent hydrolases including glyoxylases | |
| Swol_1849 | 0.00179 | Acyl carrier protein | |
| Nucleotide transport and metabolism | | |  |
| Swol_0040 | 0.00043 | dTMP kinase |  |
| Swol_1280 | 0.00024 | Oxidoreductase FAD/NAD(P)-binding | |
| Swol_1282 | 0.00024 | Carbamoyl-phosphate synthase, small subunit | |
| Swol_1779 | 0.00031 | Adenylosuccinate lyase | |
| Swol_2353 | 0.00043 | Thymidylate synthase complementing protein ThyX | |
| Cell motility | | |  |
| Swol_0532 | 0.00037 | Twitching motility protein | |
| Swol_1449 | 0.00032 | Protein-glutamate methylesterase | |
| Cell wall/membrane/envelope biogenesis | | |  |
| Swol_0228 | 0.0003 | Nucleoside-diphosphate-sugar epimerase | |
| Intracellular trafficking, secretion, and vesicular transport | | | |
| Swol_1443 | 0.00042 | Sec-independent protein secretion pathway component | |

**Supplemental Table 4. Proteins detected only during coculture growth on crotonate with the methanogen, *M. hungatei.***

| Locus Tag | NSAF | Function |
| --- | --- | --- |
| Swol_0112 | 0.00022 ± 0.00003 | Unknown function |
| Swol_0779 | 0.00025 ± 0.00005 | S-methyl-5-thioribose-1-phosphate isomerase |
| Swol_0204 | 0.00032 ± 0.00016 | Unknown function |
| Swol_1894 | 0.00038 ± 0.00009 | HD-GYP domain |
| Swol_2412 | 0.00047 ± 0.00007 | Response regulator receiver protein |
| Swol_1523 | 0.00078 ± 0.00011 | NAD(P)H-flavin oxidoreductase, putative |
| Swol_1063 | 0.00084 ± 0.00028 | Unknown function |
| Swol_2047 | 0.00159 ± 0.00088 | Alkylhydroperoxidase AhpD core |
| Swol_1572 | 0.00209 ± 0.00177 | Diadenosine tetraphosphate (Ap4A) hydrolase |

## 2.2 Supplementary Figures
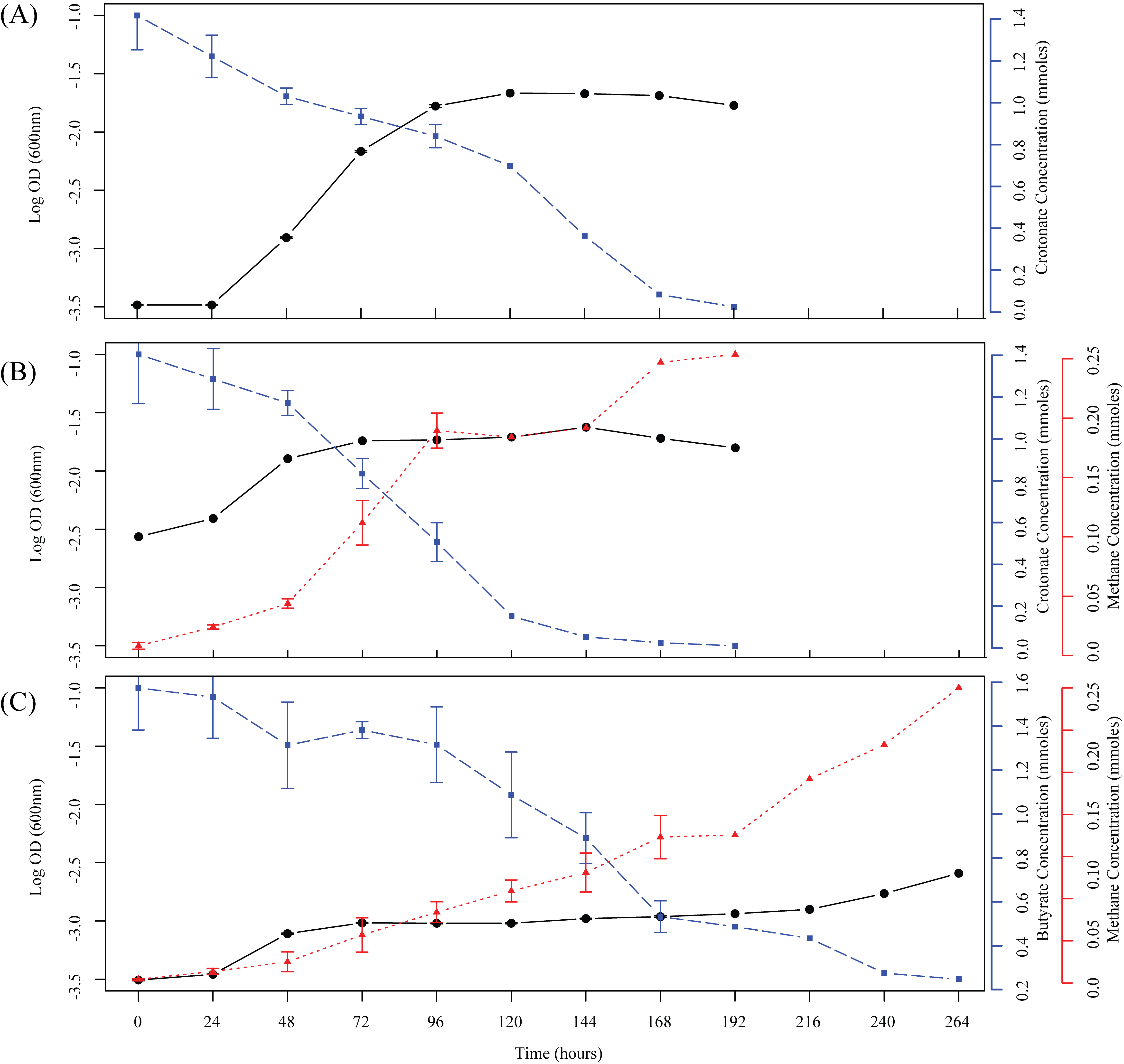


**Supplemental Figure 1. Growth of *S. wolfei* in pure culture on crotonate (A) and in coculture with *M. hungatei* on crotonate (B) and butyrate (C).** Symbols: squares, crotonate or butyrate; triangles, methane; and diamonds, absorbance. These cultures were prepared with the same inocula and media as that the respective cultures used for proteomic analyses. Means ± standard deviations of triplicate cultures are shown; error bars are shown when the standard deviations were larger than width of the symbol.

##
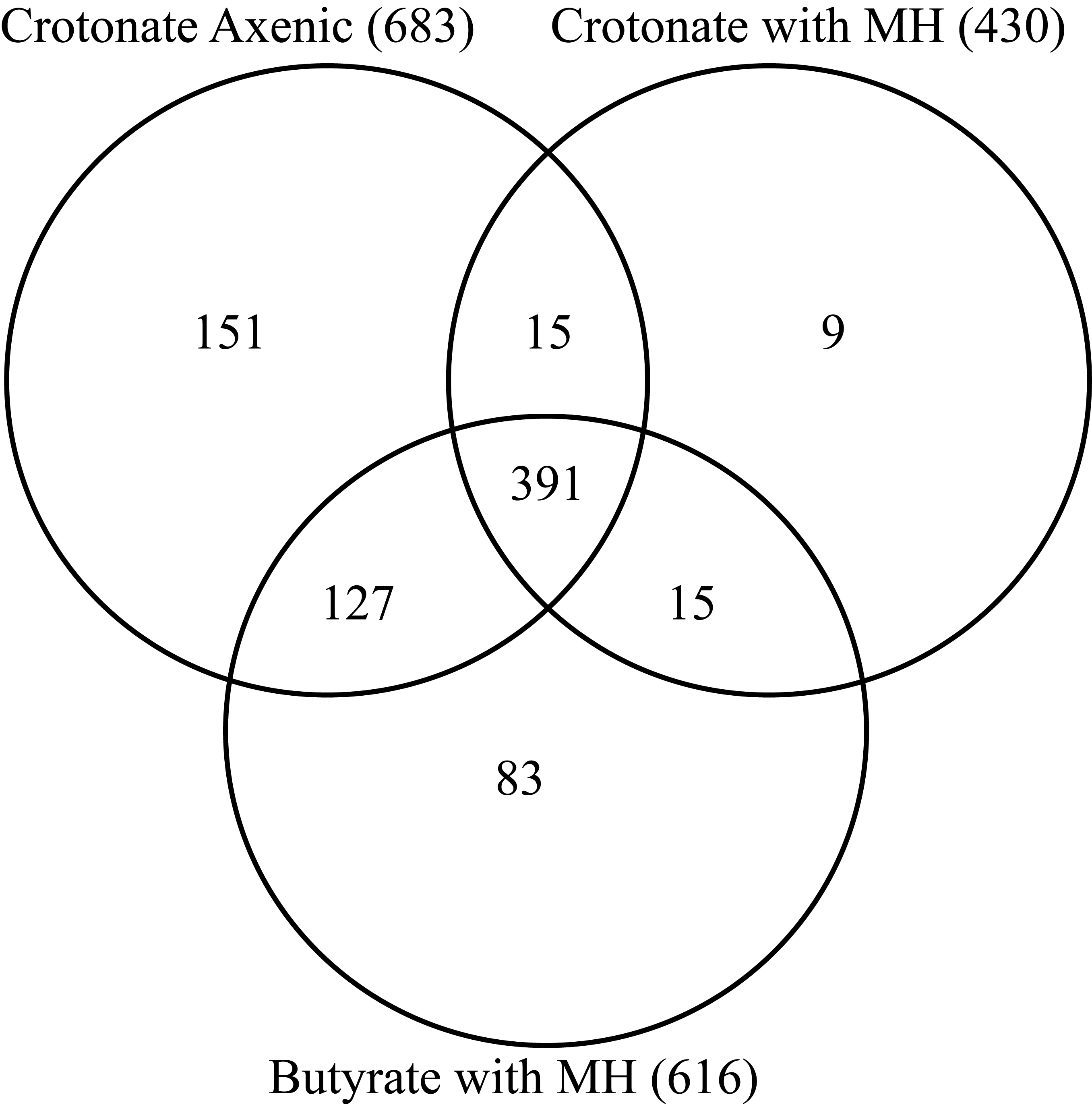


**Supplemental Figure 2. Number of proteins detected under each condition.** Abbreviation: MH, *M. hungatei*.

##
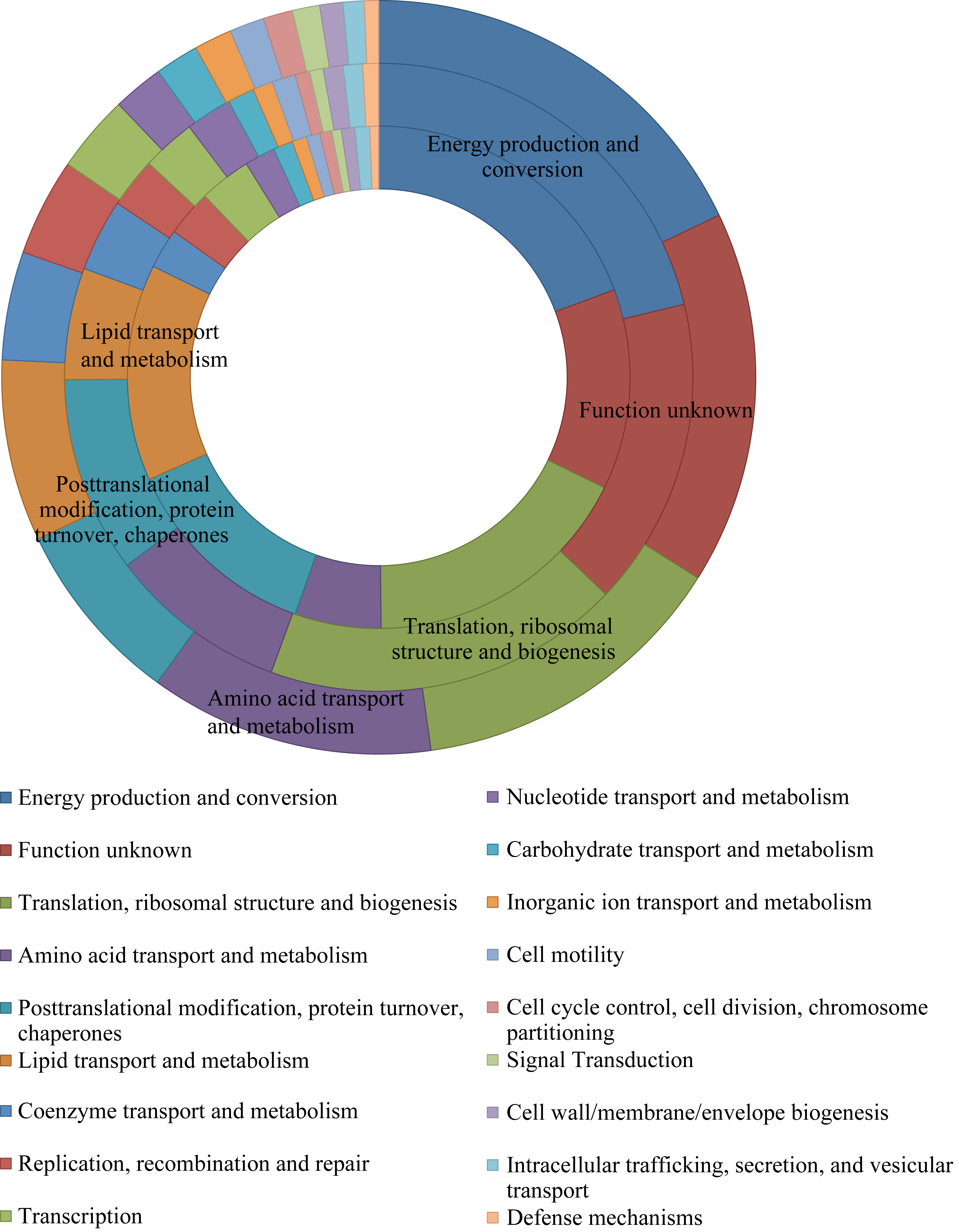


**Supplemental Figure 3. Abundance of peptides assigned to proteins in different COG functional groups for each growth conditions.**
